# Integrated chemical and radiological risk assessment of livestock products from the Abai region adjacent to the former Semipalatinsk Nuclear Test Site, Kazakhstan

**DOI:** 10.3389/fvets.2026.1830035

**Published:** 2026-07-01

**Authors:** Zhadyra Amirgalina, Ainur Serikova, Shyngys Suleimenov, Zhaxylyk Serikov, Shynar Abdykarimova, Nazgul Taukebayeva, Sergazy Duyssembayev

**Affiliations:** Department of Veterinary, Shakarim University, Semey, Kazakhstan

**Keywords:** livestock products, non-carcinogenic risk assessment, One Health, radiological risk, radionuclides, Semipalatinsk Nuclear Test Site, toxic and potentially toxic metals, veterinary public health

## Abstract

This study evaluated the chemical and radiological safety of animal-derived food products from the Abai region of Kazakhstan adjacent to the former Semipalatinsk Nuclear Test Site. A total of 450 samples of beef, horse meat, mutton, poultry meat, and whole cow’s milk were collected from the Abai, Ayagoz, and Aksuat districts, which differ in their degree of potential technogenic and residual radiological impact. Lead, cadmium, copper, and zinc concentrations were determined by anodic stripping voltammetry, while ^137^Cs and ^90^Sr activities were measured by gamma spectrometry and radiochemical beta counting. Non-carcinogenic risk was assessed using the Target Hazard Quotient and Hazard Index, and radiological risk was evaluated using the Annual Effective Dose and Excess Lifetime Cancer Risk. Significant inter-district differences were observed, with the highest contaminant burdens generally detected in the Abai district and the lowest in the Aksuat district. Lead concentrations in whole cow’s milk exceeded the applicable regulatory limit in all three districts, representing the most important food-safety finding of the study, whereas most other analytes remained within permissible levels. Despite these local exceedances, all calculated non-carcinogenic and radiological risk indicators remained below internationally accepted threshold values for adults under the assumed consumption scenario. These findings indicate a low overall health risk from consumption of the investigated products, while supporting the need for territorially differentiated and product-specific veterinary-sanitary monitoring in historically affected regions within a One Health framework.

## 1 Introduction

The Semipalatinsk Nuclear Test Site (SNTS) represents one of the most important historical sources of long-term radiological concern in Kazakhstan. Over a 40-year period, approximately 456 nuclear tests, including atmospheric, surface, and underground detonations, were conducted at the site. The cumulative yield of the nuclear charges tested between 1949 and 1963 has been reported to exceed the yield of the atomic bomb dropped on Hiroshima by approximately 2,500 times, and expert estimates indicate that approximately 1.3 million people were exposed to radiation. Although more than three decades have passed since the closure of the test site, residual contamination and related health concerns remain relevant for territories adjacent to the former SNTS (1).

Food products of animal origin are important components of the human diet and may serve as pathways for the transfer of environmental contaminants to the population. In Kazakhstan, ensuring the safety of meat and dairy products is therefore a key objective of veterinary public health and national veterinary control systems (2, 3). In areas affected by historical radiological impact and ongoing anthropogenic pressures, monitoring of radionuclides and toxic and potentially toxic metals in livestock products is particularly important for assessing food-chain safety (4, 5).

The transfer of radionuclides into milk and meat is governed by interconnected environmental and biological processes, including atmospheric deposition, soil retention, plant uptake, ingestion by livestock through contaminated feed and water, gastrointestinal absorption, and accumulation in edible tissues or secretion into milk (6–8). Among long-lived radionuclides, ^137^Cs tends to accumulate predominantly in muscle tissue, whereas ^90^Sr has a stronger affinity for bone tissue because of its chemical similarity to calcium (8, 9). These biokinetic differences influence the distribution of radionuclides among meat, milk, and other animal-derived food products.

In addition to radiological contamination, agroecosystems may be affected by toxic and potentially toxic metals. Lead (Pb) and cadmium (Cd) are priority toxic elements because of their persistence, bioaccumulative potential, and adverse toxicological effects, even under chronic low-dose exposure. Copper (Cu) and zinc (Zn) are essential trace elements required for normal physiological functions; however, they may become toxic when present at elevated concentrations (10–13). Livestock may be exposed to these elements through feed, water, soil ingestion, and local environmental sources, making veterinary monitoring essential for preventing food-chain contamination.

The Abai region of Kazakhstan is of particular interest for integrated food safety assessment because it combines the long-term radiological legacy of the former SNTS with local geochemical heterogeneity, land-use characteristics, and possible contemporary anthropogenic inputs (4, 14, 15). International experience from post-contamination regions, including areas affected by the Chernobyl and Fukushima accidents, shows that current radiological risks in food products may decline over time, while local heterogeneity in contaminant transfer can persist for decades (16–20). Therefore, assessment of livestock products from historically affected territories should consider both radionuclides and chemical contaminants within a unified veterinary public health framework.

Modern food safety assessment increasingly relies on quantitative risk indicators rather than simple comparison with maximum permissible concentrations. The target hazard quotient (THQ) and hazard index (HI) are commonly used to evaluate non-carcinogenic risk from chemical contaminants (21, 22), whereas the annual effective dose (AED) and excess lifetime cancer risk (ELCR) are used to assess radiological risk (9, 23, 24). Within the One Health framework, the present study considers the interconnected pathway from soil, water, and pasture vegetation to livestock and finally to humans through consumption of meat and whole cow’s milk. Therefore, the aim of this study was to assess the concentrations of ^137^Cs, ^90^Sr, Pb, Cd, Cu, and Zn in meat and milk produced in different districts of the Abai region and to quantify the associated non-carcinogenic and radiological risks for the adult population.

## 2 Materials and methods

### 2.1 Study design and sampling area

The study was conducted at ShakarimLab, Non-profit Joint Stock Company Shakarim University (Semey, Republic of Kazakhstan). The investigation focused on livestock products, including beef, horse meat, mutton, cow’s milk, and poultry meat, produced in three districts of the Abai region that are characterized by different degrees of potential anthropogenic impact and residual radiological exposure.

Approximate district-level geographic coordinates were used to describe the general location of the sampling territories: Abai district (administrative center Karaul, approximately 48.945° N, 79.255° E), Ayagoz district (administrative center Ayagoz, approximately 47.964° N, 80.434° E), and Aksuat district (administrative center Aksuat, approximately 47.761° N, 82.809° E). These coordinates refer to administrative district centers rather than individual farms or markets, because exact sampling-site coordinates were not recorded for confidentiality and logistical reasons.

The main radiation-risk zones are classified according to the accumulated effective radiation dose as follows: the zone of extreme radiation risk, with accumulated doses exceeding 100 rem; the zone of maximum radiation risk, from 35 to 100 rem; the zone of increased radiation risk, from 7 to 35 rem; and the zone of minimal radiation risk, from 0.1 to 7 rem.

Sampling was carried out in areas differing in radiation-risk status and distance from the former Semipalatinsk Nuclear Test Site (SNTS). The study included the Abai district, which belongs to the zone of extreme radiation risk and is located approximately 30–80 km from the former SNTS; the Ayagoz district, characterized as a zone of increased radiation risk and located approximately 300–400 km from the test site; and the Aksuat district, classified as a zone of maximum radiation risk and located approximately 600 km from the former SNTS.

This study employed a comparative cross-sectional design aimed at assessing territorial differences in contaminant burden among districts with different historical and environmental risk profiles. The classification of the districts as differing in potential technogenic and residual radiological impact was based on their radiation-risk status, distance from the former SNTS, and historical exposure background. Therefore, the comparison was designed to evaluate whether radionuclide and metal concentrations in livestock products differed among territories with contrasting environmental risk characteristics.

### 2.2 Sample collection and size

The sample size in the present study (*n* = 450) was determined to provide balanced coverage of different categories of products of animal origin and territorial zones. In each of the three administrative districts included in the study—Abai, Ayagoz, and Aksuat—30 samples were collected for each of five product categories: beef, horse meat, mutton, poultry meat, and whole cow’s milk. Thus, the total sample size was calculated as follows: 30 × 5 × 3 = 450.

This sampling structure ensured an equal distribution of samples across product types and study districts, allowing comparative assessment of contamination levels among territories with different degrees of potential anthropogenic and residual radiological impact.

The sampling strategy was purposive-stratified rather than fully random. Samples were stratified by district and product category, while specific private farms and local markets were selected based on accessibility, availability of the required product types, volume of product turnover, and representativeness for local supply chains. Therefore, the study provides a balanced comparative assessment across districts and product categories; however, the possibility of selection bias related to site accessibility and product availability cannot be completely excluded.

The study covered the period from April to June 2025 and therefore did not account for seasonal variability. It is known that radionuclide and toxic and potentially toxic metal levels in products of animal origin may vary seasonally due to differences in animal feeding practices, such as pasture grazing in summer and stored feed use in winter, as well as changes in hydrological conditions and soil biogeochemical processes. Accordingly, the results should be interpreted as reflecting the spring-early summer period rather than year-round contamination patterns.

Sample collection was performed in accordance with current veterinary and sanitary requirements for the sampling of food products of animal origin. Samples were transported under controlled conditions, and sample processing was conducted according to standardized laboratory protocols.

### 2.3 Determination of radionuclides

The activity concentration of ^137^Cs was measured in accordance with the certified measurement procedure MVI. MN 4727-2013 using gamma spectrometry. Measurements were performed with a NaI(Tl) scintillation detector (63 × 63 mm) on an RKG-AT1320C gamma radiometer (ATOMTEX, Republic of Belarus). Prior to measurement, all samples were subjected to standard sample preparation procedures. Range from ^137^Cs − 3.7 to 10^6^Bk/kg, measurement error was not more than 10–20%.

The determination of ^90^Sr was performed after radiochemical separation followed by beta counting using a TRI-CARB 2900TR liquid scintillation beta spectrometer.

The specific activity of ^90^S in samples of animal products (meat, cow’s milk) was determined by the radiometric method after strict radiochemical separation and concentration. Radiochemical analysis of ^90^Sr. Radiochemical separation: The meat/milk cinder has been dissolved in concentrated HNO_3_ with the addition of stable carrier Sr^2+.^ Primary concentration was carried out by the co-deposition of alkaline earth metal oxalates with addition of (NH_4_)C_2_O_4_ (pH4.0–4.5). The removal of calcium and magnesium from excess was carried out by a cycle of gassing and redeposition in the environment of 100% HNO_3_ nitric acid. Elimination of interference ^90^Y: Present in the sample of daughter ^90^Y were removed by the co-sedimentation with iron hydroxide Fe(OH)_3_ at pH8.0. The moment of separation of precipitation was recorded as “zero time” (t_0_). The purified solution lasted 14–18 days until reaching a centuries-old radioactive equilibrium between ^90^Sr and the newly accumulated ^90^Y. Beta score and separation ^89^Sr: Measurements were performed on the high energy emission of the daughter ^90^Y (Emax = 2.28MеV) at the liquid scintillation spectrometer Tri-Carb 2900TR (PerkinElmer, United States) with Ultima Gold LLT. Interference from the possible presence of ^89^Sr (Emax = 1.49 MеV) was eliminated by a two-channel calculation method in different energy windows: 0–1.4 MеV (total signal) and 1.5–2.3 MеV (only peak ^90^Y) with mathematical separation of instrument deposits.

The relative measurement uncertainty ranged from 15 to 25%. The method detection limit (LOD) was approximately 0.1 Bq/kg for meat samples and 0.05 Bq/L for milk samples, calculated using the 3σ criterion based on blank/control samples. Limits of quantification (LOQ) were calculated as ten times the standard deviation of blank measurements and were used to verify the reliability of quantified activity concentrations. Activity concentrations below the LOD were reported as <LOD.

To ensure the reliability and reproducibility of the analytical results, a quality assurance and quality control (QA/QC) system was implemented in the present study in accordance with generally accepted requirements for the analysis of food products. For the determination of radionuclides (^137^Cs and ^90^Sr), validated gamma-spectrometric and radiochemical methods combined with liquid scintillation spectrometry were applied. Instrument calibration was performed using certified radioactive standard sources traceable to reference standards. Measurement stability was monitored through the regular analysis of control samples and background/blank samples. Certified reference materials for all analyzed food matrices were not available; therefore, analytical performance was verified using calibration standards, blank samples, control samples, and repeated measurements.

### 2.4 Determination of toxic and potentially toxic metals and QA/QC

The mass fractions of cadmium Cd, lead Pb, copper Cu, and zinc Zn in the livestock and food samples were determined by anodic stripping voltammetry (ASV). The measurements were executed in strict accordance with the certified methodology FR 1.31.2008.01733 (developed by CJSC “Aquilon,” Russian Federation; Certificate of Metrological Certification No. 22-08 dated March 4, 2008).

The electrochemical system utilized a three-electrode cell configuration comprising: Working (indicator) electrode: a glassy carbon electrode (model AKU-1); Reference electrode: a saturated silver/silver chloride electrode Ag/AgCl filled with a saturated potassium chloride KCl solution; Auxiliary (counter) electrode: a glassy carbon crucible, which simultaneously served as the electrochemical measurement cell.

The deposition parameters for the electrochemical accumulation of metals were set at a deposition potential of −1.2 V vs. Ag/AgCl. The deposition time ranged from 10 to 20 s under automated continuous solution stirring using the instrument’s built-in magnetic stirrer. The stripping (dissolution) step and subsequent recording of voltammetric curves were performed in the square-wave voltammetry (SWV) mode. This mode represents the standard operating procedure for the AKV-07MK analyzer, ensuring an optimal analytical signal-to-background current ratio.

The operational limits of detection (LOD, at a signal-to-noise ratio of S/N = 3) and limits of quantification (LOQ, at S/N = 10) for the investigated food matrices were established as follows: Lead (Pb): LOD = 0.002 mg/kg; LOQ = 0.005 mg/kg (Note: the lower limit of the methodology’s measurement range for lead is 0.0020 mg/kg). Cadmium (Cd): LOD = 0.005 mg/kg; LOQ = 0.020 mg/kg (Note: the lower limit of the methodology’s measurement range for cadmium is 0.020 mg/kg). Copper (Cu): LOD = 0.001 mg/kg; LOQ = 0.003 mg/kg (Note: the lower limit of the methodology’s measurement range for copper is 0.0010 mg/kg). Zinc (Zn): LOD = 0.005 mg/kg; LOQ = 0.010 mg/kg (Note: the lower limit of the methodology’s measurement range for zinc is 0.010 mg/kg).

Sample preparation for meat and milk was performed in accordance with GOST 26929-94: Briefly, 20 g of homogenized sample was taken and subjected to gradual drying at temperatures up to 150 °C, followed by carbonization and ashing at temperatures up to 450 °C. The ash residue was then treated with concentrated nitric acid, followed by repeated ashing. The final ash was dissolved in hydrochloric acid and diluted to a final volume of 25 mL. Calibration linearity was verified within the relevant concentration ranges, with coefficients of determination of *R*^2^ ≥ 0.995.

Method accuracy was assessed using spike-recovery tests. Known amounts of the target elements were added to control samples, and recovery percentages were subsequently calculated. Recovery values ranged from 85 to 110% for all analyzed elements, which was considered acceptable for analytical accuracy. Certified reference materials for all matrices were not available; therefore, method accuracy was evaluated using spike-recovery tests and certified standard solutions.

Precision was evaluated by analyzing replicate samples (*n* ≥ 3). The relative standard deviation (RSD) did not exceed 10% for most determinations. The limits of detection (LOD) and limits of quantification (LOQ) were calculated as three and ten times the standard deviation of blank samples, respectively.

Additional quality control measures included the analysis of blank samples to exclude possible contamination, regular verification of instrument sensitivity, monitoring of signal drift over time, and strict adherence to standardized sample preparation procedures to minimize matrix effects. Although interlaboratory validation was not performed within the scope of this study, the methods used were based on recognized analytical protocols and are widely applied in the toxicological analysis of food products.

Overall, the implemented QA/QC procedures confirmed the reliability, accuracy, and reproducibility of the analytical data obtained in this study.

### 2.5 Non-carcinogenic risk assessment

Non-carcinogenic risk associated with dietary exposure to Pb, Cd, Cu, and Zn was assessed using the Target Hazard Quotient (THQ) in accordance with USEPA guidance:


$$\mathrm{THQ}=\frac{\left(C\times \mathrm{IR}\right)}{\left(\mathrm{RfD}\times \mathrm{BW}\right)}$$

Where *C* is the mean concentration of the analyte in the food product (mg/kg for meat and mg/L for milk), *IR* is the daily ingestion rate, *RfD* is the oral reference dose, and *BW* is body weight. The Hazard Index (HI) was calculated as the sum of the individual THQ values for the four elements.

For adults, BW was set at 70 kg, which is commonly used as a standard adult body weight in deterministic health risk assessment models (10). The daily food ingestion rates IR were calculated based on the official data of the Bureau of National Statistics of the Agency for Strategic Planning and Reforms of the Republic of Kazakhstan for household food consumption. For the adult population, the synchronized ingestion rates were set at 0.192 kg/day for meat and 0.548 L/day for milk, which strictly correspond to the annual intake volumes of 70 kg/year and 200 L/year, respectively, used in the radiological assessment section. The oral reference doses (mg/kg body weight/day) were 0.0035 for Pb, 0.001 for Cd, 0.04 for Cu, and 0.30 for Zn (10, 21, 22). THQ < 1 and HI < 1 were interpreted as indicating no significant non-carcinogenic risk under the assumed adult exposure scenario.

Carcinogenic risk for Pb and Cd was not calculated in the present study because the assessment focused on non-carcinogenic dietary exposure using oral reference doses and THQ/HI indicators. In addition, internationally harmonized oral cancer slope factors applicable to the specific food ingestion scenario and local population parameters were not available within the adopted assessment framework. Therefore, the absence of carcinogenic risk calculations for toxic metals is acknowledged as a limitation of the study.

The present risk assessment was performed for adults because reliable local age-specific consumption data for children were not available for the studied districts. Children may have higher exposure per unit body weight and greater sensitivity to Pb and Cd; therefore, the obtained THQ and HI values should not be directly extrapolated to children. Separate child-specific exposure scenarios should be considered in future studies when appropriate dietary data become available.

### 2.6 Radiological risk assessment

Radiological risk was evaluated using the Annual Effective Dose (AED) and Excess Lifetime Cancer Risk (ELCR). AED was calculated as:


AED=A×CR×DCF

Where *A* is radionuclide activity concentration (Bq per unit of product), *CR* is the annual consumption rate, and *DCF* is the adult ingestion dose coefficient.

The annual consumption rates applied in the calculations were 70 kg/year for meat products and 200 L/year for whole cow’s milk. Adult ingestion dose coefficients of 1.3 × 10^−8^ Sv/Bq for ^137^Cs and 2.8 × 10^−8^ Sv/Bq for ^90^Sr were taken from the ICRP dose coefficient compendium for adult members of the public (25).

ELCR was calculated as ELCR = AED × 70 × 0.05, where AED values expressed in mSv/year were converted to Sv/year before calculation, 70 years is the assumed lifetime, and 0.05 Sv^−1^ is the nominal lifetime cancer risk coefficient recommended by the ICRP (9). The 70-year lifetime and 70 kg adult body weight were used as standard deterministic exposure assumptions in accordance with commonly applied health risk assessment practice (10).

### 2.7 Statistical analysis

Statistical analysis was performed using IBM SPSS Statistics version 26.0. Results are presented as mean ± standard deviation (M ± SD). Interdistrict differences were assessed using one-way ANOVA followed by Tukey’s *post hoc* test. Statistical significance was set at *p* < 0.05.

## 3 Results

### 3.1 Radionuclide content in meat and milk

The activity concentrations of ^137^Cs and ^90^Sr were measured in beef, horse meat, mutton, poultry meat, and cow’s milk samples collected from three districts of the Abai region of Kazakhstan (Table 1).

**Table 1:** Activity concentrations of ^137^Cs and ^90^Sr in meat and milk samples collected from different districts of the Abai region.

District	Product	^137^Cs activity	^90^Sr activity
Abai	Beef	18.16 ± 1.02 a	0.94 ± 0.24 a
	Horse meat	15.53 ± 0.81 a	0.80 ± 0.16 a
	Mutton	17.34 ± 0.93 a	0.90 ± 0.18 a
	Poultry	12.01 ± 0.42 a	<LOD
	Milk	11.66 ± 0.21 a	2.20 ± 0.09 a
Ayagoz	Beef	12.07 ± 0.38 b	0.62 ± 0.14 b
	Horse meat	10.95 ± 0.27 b	0.57 ± 0.12 b
	Mutton	9.41 ± 0.21 b	0.49 ± 0.10 b
	Poultry	5.64 ± 0.19 b	<LOD
	Milk	5.00 ± 0.12 b	1.46 ± 0.04 b
Aksuat	Beef	5.54 ± 0.17 c	0.29 ± 0.09 c
	Horse meat	4.07 ± 0.15 c	0.21 ± 0.07 c
	Mutton	4.88 ± 0.16 c	0.25 ± 0.08 c
	Poultry	3.61 ± 0.11 c	<LOD
	Milk	3.63 ± 0.07 c	1.20 ± 0.02 c

Values are presented as mean ± standard deviation (M ± SD). For each product category, *n* = 30 samples were analyzed per district. For meat samples, radionuclide activity concentrations are expressed as Bq/kg; for milk samples, as Bq/L. Different superscript letters (a–c) within the same product category indicate statistically significant differences among districts, as determined by one-way ANOVA followed by Tukey’s post hoc test (*p* < 0.05). Activity concentrations below the limit of detection are reported as <LOD. The reference limit for ^137^Cs in food products was 200 Bq/kg according to applicable sanitary regulations and TR TS 021/2011 “On Food Safety”.

The analysis of radionuclide activity concentrations (^137^Cs and ^90^Sr) in meat and milk samples produced in different districts of the Abai region revealed clear spatial heterogeneity and statistically significant interdistrict differences.

The highest radionuclide activity concentrations were recorded in samples from the Abai district. In meat products, ^137^Cs activity ranged from 12.01 ± 0.42 Bq/kg in poultry meat to 18.16 ± 1.02 Bq/kg in beef. In milk samples from the same district, the activity concentrations of ^137^Cs and ^90^Sr were 11.66 ± 0.21 Bq/L and 2.20 ± 0.09 Bq/L, respectively, representing the highest values observed in the study.

Intermediate radionuclide levels were detected in the Ayagoz district. The activity concentration of ^137^Cs in meat products ranged from 5.64 to 12.07 Bq/kg, whereas in milk it was 5.00 ± 0.12 Bq/L. The activity concentration of ^90^Sr in milk decreased to 1.46 ± 0.04 Bq/L. The lowest radionuclide concentrations were observed in the Aksuat district, where ^137^Cs levels in meat products did not exceed 5.54 Bq/kg, and the activity concentration in milk was 3.63 ± 0.07 Bq/L.

Product-specific differences in radionuclide accumulation were also observed. Across all districts, ^137^Cs activity in meat products generally followed the same pattern: beef > mutton > horse meat > poultry meat. The activity concentration of ^90^Sr in poultry meat was below the limit of detection in all analyzed samples. In contrast, ^90^Sr concentrations were higher in milk than in meat products, reaching 2.20 Bq/L in milk compared with 0.94 Bq/kg in beef from the Abai district.

A statistically significant decrease in radionuclide activity concentrations (*p* < 0.05) was observed among the studied districts, with the highest values in the Abai district and the lowest values in the Aksuat district. This pattern suggests a general spatial trend associated with differences in radiation-risk status and distance from the former Semipalatinsk Nuclear Test Site. However, the observed pattern should be interpreted as a general interdistrict tendency rather than a strictly uniform gradient for all radionuclide-product combinations.

Despite the observed interdistrict differences, the activity concentrations of ^137^Cs in all analyzed samples remained far below the maximum permissible level of 200 Bq/kg. Thus, with respect to the radionuclides analyzed, the investigated products did not exceed the established permissible levels; however, continued monitoring remains important given the spatial variability observed among the districts.

### 3.2 Radiological risk assessment from meat and milk consumption

The observed spatial differences in ^137^Cs and ^90^Sr activity concentrations were consistent with the results of the radiological risk assessment. The calculated annual effective dose (AED) and excess lifetime cancer risk (ELCR) associated with the consumption of meat and milk are presented in Table 2.

**Table 2:** Annual effective dose (AED) and excess lifetime cancer risk (ELCR) associated with meat and milk consumption.

District	Product	^137^Cs activity	^90^Sr activity	AED (^137^Cs), mSv/year	AED (^90^Sr), mSv/year	Total AED, mSv/year	ELCR (×10^−5^)
Abai	Beef	18.16	0.94	0.0165	0.00184	0.0183	6.41
	Horse meat	15.53	0.80	0.0141	0.00157	0.0157	5.50
	Mutton	17.34	0.90	0.0158	0.00176	0.0176	6.16
	Poultry	12.01	<LOD	0.0109	<LOD	0.0109	3.8
	Milk	11.66	2.20	0.0303	0.0123	0.0426	14.9
Ayagoz	Beef	12.07	0.62	0.0110	0.00121	0.0122	4.27
	Horse meat	10.95	0.57	0.0100	0.00112	0.0111	3.89
	Mutton	9.41	0.49	0.0086	0.00096	0.0096	3.36
	Poultry	5.64	<LOD	0.0051	<LOD	0.0051	1.8
	Milk	5.00	1.46	0.0130	0.0082	0.0212	7.42
Aksuat	Beef	5.54	0.29	0.0050	0.00057	0.0056	1.96
	Horse meat	4.07	0.21	0.0037	0.00041	0.0041	1.43
	Mutton	4.88	0.25	0.0044	0.00049	0.0049	1.71
	Poultry	3.61	<LOD	0.0033	<LOD	0.0033	1.2
	Milk	3.63	1.20	0.0095	0.0067	0.0162	5.16

* AED, Annual Effective Dose of internal exposure; ELCR, Excess Lifetime Cancer Risk. For each product category, *n* = 30 samples were analyzed per district. For meat samples, radionuclide activity concentrations are expressed as Bq/kg; for milk samples, as Bq/L. ^90^Sr was below the limit of detection in poultry meat samples. Risk calculations were performed for the adult population using average annual consumption rates of 70 kg/year for meat products and 200 L/year for whole cow’s milk. The total annual effective dose (AED) was calculated as the sum of the contributions from ^137^Cs and ^90^Sr. AED values expressed in mSv/year were converted to Sv/year before ELCR calculation. The excess lifetime cancer risk (ELCR) was calculated based on the total AED, an assumed lifetime duration of 70 years, and a nominal cancer risk factor of 0.05 Sv^−1^.

The estimated annual effective dose of internal exposure associated with meat consumption showed that the contribution of ^137^Cs ranged from 0.0033 to 0.0165 mSv/year, whereas the contribution of ^90^Sr ranged from 0.00041 to 0.00184 mSv/year. The highest total AED values among meat products were recorded in the Abai district, particularly for beef and mutton, whereas the lowest values were observed in the Aksuat district. In all cases, the total AED from meat consumption was substantially below the reference dose limit of 1 mSv/year for the general public.

In poultry meat samples, the activity of ^90^Sr was below the method detection limit; therefore, the estimated AED for this product category was determined solely by the contribution of ^137^Cs. The AED values associated with poultry meat consumption ranged from 0.0033 to 0.0109 mSv/year, with the highest value observed in the Abai district and the lowest in the Aksuat district.

For whole milk, the total AED ranged from 0.0162 to 0.0426 mSv/year. The highest value was found for milk from the Abai district, which was associated with higher activity concentrations of both ^137^Cs and ^90^Sr. Nevertheless, all calculated AED values for milk also remained below the reference level of 1 mSv/year.

The estimated excess lifetime cancer risk associated with the consumption of the investigated products ranged from 1.20 × 10^−5^ to 14.9 × 10^−5^ The lowest risk was associated with the consumption of poultry meat from the Aksuat district, whereas the highest risk was associated with milk consumption from the Abai district. These values indicate a low estimated level of radiological risk. However, the higher ELCR values observed for milk highlight the need for continued monitoring of dairy products in areas adjacent to the former Semipalatinsk Nuclear Test Site.

The analysis of the relative contribution of individual radionuclides showed that, in meat products, ^137^Cs was the main contributor to the total AED, accounting for approximately 89–91% of the total dose, whereas the contribution of ^90^Sr generally did not exceed 1–11%. In poultry meat, the contribution of ^137^Cs was 100%, as ^90^Sr was not detected.

In contrast to meat products, milk showed a noticeably higher relative contribution of ^90^Sr to the total AED. Its contribution ranged from approximately 29 to 42% of the total dose, which may be related to the chemical similarity of strontium to calcium and its more pronounced transfer into dairy products. Thus, ^137^Cs remained the dominant dose-forming radionuclide in meat products, whereas the contribution of ^90^Sr was substantially higher in milk.

Quantitative analysis of radionuclide contributions indicates that internal radiation exposure associated with the consumption of meat products in the studied districts was predominantly attributable to ^137^Cs. Across all districts and meat types, ^137^Cs accounted for approximately 89–100% of the total annual effective dose, whereas the contribution of ^90^Sr did not exceed 11%.

For beef, horse meat, and mutton, the contribution of ^137^Cs remained within a narrow range, from 89.3 to 90.2%, indicating its dominant role in the formation of the dose burden from meat products. The contribution of ^90^Sr for these meat types was relatively low, ranging from approximately 9.8 to 10.7%.

The most pronounced predominance of ^137^Cs was observed in poultry meat, where its contribution reached 100%. This was due to the fact that ^90^Sr activity in poultry meat samples was below the limit of detection; therefore, its contribution to the total dose was not registered.

These findings reflect differences in the biokinetic behavior of the radionuclides. ^137^Cs is characterized by relatively high mobility and a tendency to accumulate in muscle tissue, which represents the main edible fraction of meat products. In contrast, ^90^Sr has a strong affinity for bone tissue, which limits its transfer to muscle fractions and reduces its contribution to the dose associated with meat consumption.

A different pattern was observed for milk. The contribution of ^137^Cs decreased from the Abai district to the Aksuat district, from 71.2% in Abai to 61.3% in Ayagoz and 58.4% in Aksuat. At the same time, the contribution of ^90^Sr increased from 28.8 to 41.6%. This indicates a more substantial role of ^90^Sr in dose formation from milk consumption compared with meat products (Table 3).

**Table 3:** Contribution of ^137^Cs and ^90^Sr to total annual effective dose from meat and milk consumption (%).

District	Product	Contribution ^137^Cs (%)	Contribution ^90^Sr (%)
Abai	Beef	90.2	9.8
	Horse meat	89.8	10.2
	Mutton	89.7	10.3
	Poultry	100	0
	Milk	71.2	28.8
Ayagoz	Beef	90.1	9.9
	Horse meat	90.0	10.0
	Mutton	89.6	10.4
	Poultry	100	0
	Milk	61.3	38.7
Aksuat	Beef	89.3	10.7
	Horse meat	90.2	9.8
	Mutton	89.8	10.2
	Poultry	100	0
	Milk	58.4	41.6

Thus, the radiological risk associated with the consumption of meat products in the studied districts was mainly determined by ^137^Cs, whereas ^90^Sr played a secondary role. However, in milk, the contribution of ^90^Sr was more pronounced, particularly in the Aksuat district, where it reached 41.6% of the total annual effective dose.

### 3.3 Toxic metal concentrations and non-carcinogenic risk

The concentrations of toxic elements, including lead (Pb) and cadmium (Cd), and essential elements with potential toxicity at elevated concentrations, including copper (Cu) and zinc (Zn), were analyzed in meat samples from livestock and poultry, as well as in cow’s milk, collected from the Abai, Ayagoz, and Aksuat districts (Table 4).

**Table 4:** Concentrations of toxic and potentially toxic elements in meat and milk samples.

District	Product	Pb	Cd	Cu	Zn
Abai	Beef	0.20 ± 0.04 a	0.135 ± 0.029 a	2.00 ± 0.30 a	4.24 ± 0.37 a
	Horse meat	0.18 ± 0.02 a	0.100 ± 0.025 a	1.85 ± 0.06 a	4.30 ± 0.55 a
	Mutton	0.23 ± 0.04 a	0.140 ± 0.032 a	1.80 ± 0.03 a	4.25 ± 0.30 a
	Poultry	0.21 ± 0.05 a	0.025 ± 0.011 b	1.71 ± 0.56 a	5.88 ± 0.93 a
	Milk	0.272 ± 0.068 a	0.020 ± 0.005 a	0.346 ± 0.087 ab	4.30 ± 1.08 a
Ayagoz	Beef	0.10 ± 0.03 b	0.040 ± 0.013 b	1.30 ± 0.02 b	3.72 ± 0.20 b
	Horse meat	0.11 ± 0.02 b	0.105 ± 0.017 b	1.55 ± 0.02 b	3.85 ± 0.25 b
	Mutton	0.11 ± 0.01 b	0.050 ± 0.015 b	1.45 ± 0.02 b	4.11 ± 0.35 b
	Poultry	0.11 ± 0.03 b	0.038 ± 0.013 b	2.15 ± 0.54 a	4.17 ± 0.59 b
	Milk	0.178 ± 0.045 b	0.022 ± 0.006 a	0.374 ± 0.094 a	4.37 ± 1.09 a
Aksuat	Beef	0.09 ± 0.01 c	0.025 ± 0.011 c	1.25 ± 0.02 c	3.60 ± 0.15 c
	Horse meat	0.123 ± 0.015 c	0.020 ± 0.009 c	1.10 ± 0.01 c	3.40 ± 0.20 c
	Mutton	0.08 ± 0.02 c	0.035 ± 0.012 c	1.20 ± 0.03 c	3.40 ± 0.10 c
	Poultry	0.12 ± 0.04 b	0.028 ± 0.011 c	1.61 ± 0.40 b	4.10 ± 0.30 b
	Milk	0.134 ± 0.034 c	0.024 ± 0.006 a	0.180 ± 0.045 b	4.10 ± 1.03 a

Values are presented as mean ± standard deviation (M ± SD). For meat samples, concentrations are expressed as mg/kg; for milk samples, as mg/L. Different superscript letters (a–c) within the same product category and element indicate statistically significant differences among districts according to one-way ANOVA followed by Tukey’s post hoc test (*p* < 0.05). The maximum permissible concentration for Pb in milk was 0.1 mg/L according to TR TS 021/2011 “On Food Safety”.

Lead (Pb) concentrations in whole cow’s milk exceeded the maximum permissible level of 0.1 mg/L in all three districts. The highest concentration was recorded in the Abai district (0.272 ± 0.068 mg/L), exceeding the regulatory limit by approximately 2.7-fold. In the Ayagoz and Aksuat districts, Pb concentrations were 0.178 ± 0.045 mg/L and 0.134 ± 0.034 mg/L, respectively, also exceeding the permissible threshold. Although the calculated THQ and HI values remained below 1.0 under the adult exposure scenario, the observed exceedance indicates regulatory non-compliance and requires enhanced veterinary and sanitary monitoring, particularly because milk is a staple food and may contribute substantially to chronic Pb intake. The concentrations of cadmium (Cd), copper (Cu), and zinc (Zn) in milk remained below the established regulatory limits in all districts. The highest Cu concentration in milk was observed in the Ayagoz district (0.374 ± 0.094 mg/L), while the highest Zn levels were recorded in the Abai and Ayagoz districts, ranging from 4.30 to 4.37 mg/L.

Elevated levels of Pb and Cd were also detected in meat samples, particularly in products from the Abai district. Pb concentrations in beef and mutton from the Abai district reached 0.20–0.23 mg/kg, while Cd concentrations ranged from 0.135 to 0.140 mg/kg, exceeding the values recorded in the other districts. Among the meat products, poultry from the Abai district showed a relatively high Zn concentration (5.88 ± 0.93 mg/kg), whereas the highest Cu concentration was observed in poultry from the Ayagoz district (2.15 ± 0.54 mg/kg).

In the Ayagoz and Aksuat districts, Pb and Cd concentrations in meat products were substantially lower, indicating a lower technogenic burden and a comparatively safer level of consumption for the population.

The non-carcinogenic health risk associated with meat and milk consumption was assessed using the target hazard quotient (THQ) for individual elements and the integrated hazard index (HI). The calculated THQ and HI values are presented in Table 5.

**Table 5:** Non-carcinogenic risk indicators (THQ and HI) for toxic and potentially toxic metals associated with meat and milk consumption.

District	Product	THQ (Pb)	THQ (Cd)	THQ (Cu)	THQ (Zn)	HI
Abai	Beef	0.156	0.412	0.137	0.038	0.743
	Horse meat	0.135	0.306	0.127	0.038	0.606
	Mutton	0.175	0.428	0.122	0.038	0.763
	Poultry	0.167	0.076	0.116	0.051	0.410
	Milk	0.608	0.153	0.068	0.111	0.940
Ayagoz	Beef	0.078	0.122	0.089	0.034	0.323
	Horse meat	0.082	0.319	0.106	0.034	0.541
	Mutton	0.082	0.152	0.099	0.036	0.369
	Poultry	0.084	0.176	0.078	0.111	0.769
	Milk	0.404	0.16	0.07	0.10	0.69
Aksuat	Beef	0.065	0.076	0.084	0.032	0.257
	Horse meat	0.096	0.055	0.074	0.030	0.255
	Mutton	0.063	0.108	0.082	0.030	0.283
	Poultry	0.095	0.086	0.112	0.036	0.329
	Milk	0.302	0.187	0.034	0.111	0.634

Risk calculations were performed assuming an average daily meat consumption rate of 0.192 kg/day and a whole milk consumption rate of 0.548 L/day, which strictly correspond to the regional annual intake volumes of 70 kg/year and 200 L/year, respectively, for an adult body weight of 70 kg. All calculated Target Hazard Quotient (THQ) values for individual toxic metals remained below the screening safety threshold of 1.0. Product-specific Hazard Index (HI) values ranged from 0.255 (horse meat in the Aksuat district) to 0.940 (whole milk in the Abai district), indicating no significant cumulative non-carcinogenic health risks under the evaluated exposure scenarios.

As summarized in Table 5, the integrated health risk assessment revealed that all calculated THQ values for individual toxic and potentially toxic metals across all food matrices were strictly below the critical safety threshold of 1.0. This indicates that the consumption of meat products and milk from the studied districts does not pose an isolated, single-element non-carcinogenic risk to the adult population.

However, the cumulative exposure evaluation expressed via the Hazard Index (HI) showed substantial spatial and product-specific variations. The product-specific HI values ranged from a minimum of 0.255 for horse meat in the Aksuat district to a maximum of 0.940 for whole cow milk in the Abai district. Among the meat categories, the highest cumulative risks were observed for mutton (0.763) and beef (0.743) sourced from the Abai district, driven primarily by higher baseline concentrations of Pb and Cd in these sub-regions. Cadmium was identified as the predominant contributor to the overall non-carcinogenic risk in most meat product categories.

**Figure 1:**
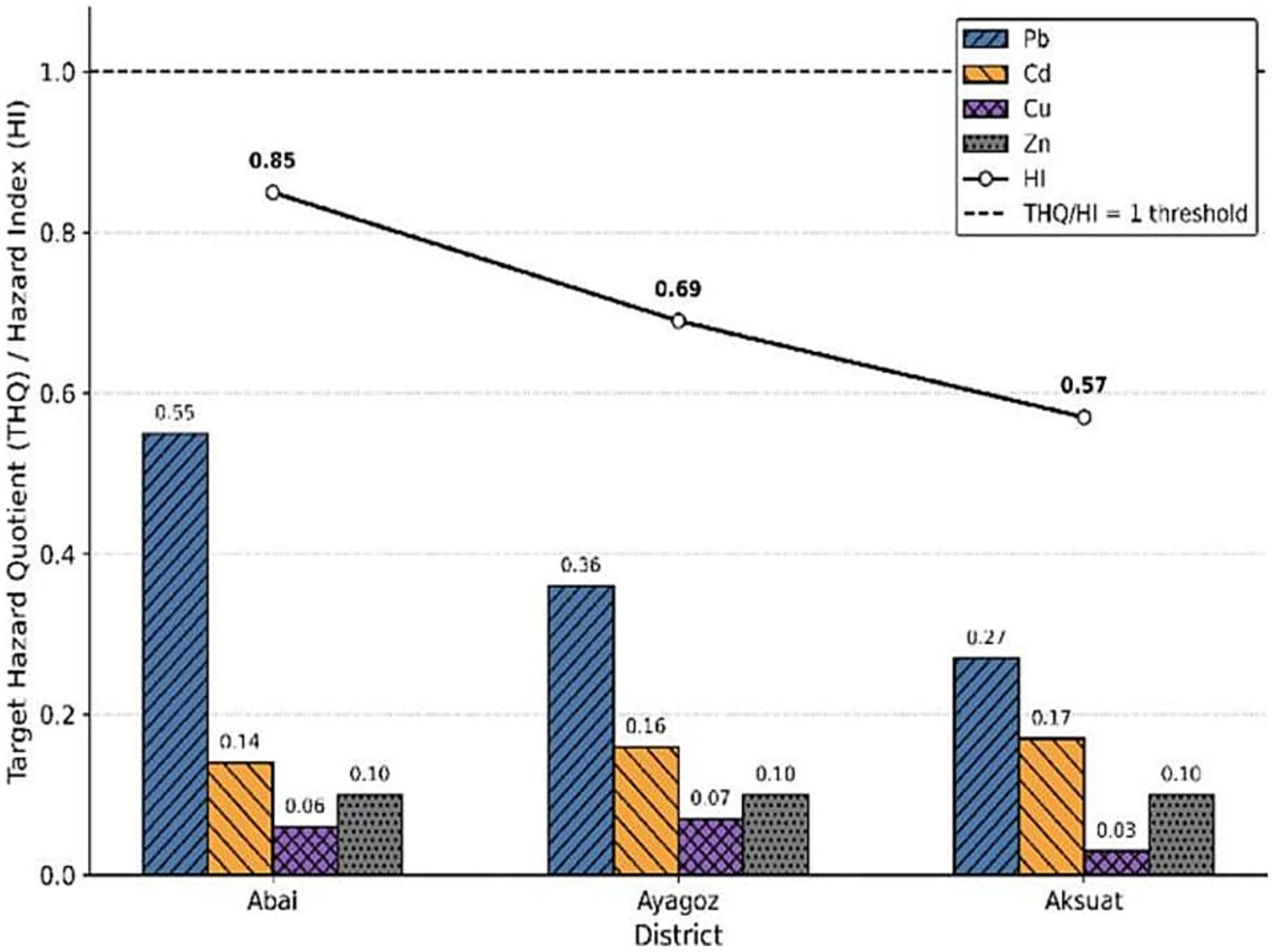
Target hazard quotient values for Pb, Cd, Cu, and Zn in whole cow’s milk from the Abai, Ayagoz, and Aksuat districts. The dashed line indicates THQ = 1, the conventional screening threshold for potential non-carcinogenic concern.

THQ calculations for whole cow’s milk demonstrated that Pb made the largest contribution to milk-related non-carcinogenic risk, followed by Cd, whereas Cu and Zn contributed less substantially. Figure 1 confirms that all milk-specific THQ values remained below the critical threshold of 1.0, although the Pb-related THQ was consistently the dominant driver, peaking in the Abai district. Notably, although the cumulative HI for milk in the Abai district neared the safety limit (0.940), no single product or district-specific matrix exceeded the threshold value of 1.0, signifying that current adult dietary exposure levels remain within the toxicologically acceptable range.

**Figure 2:**
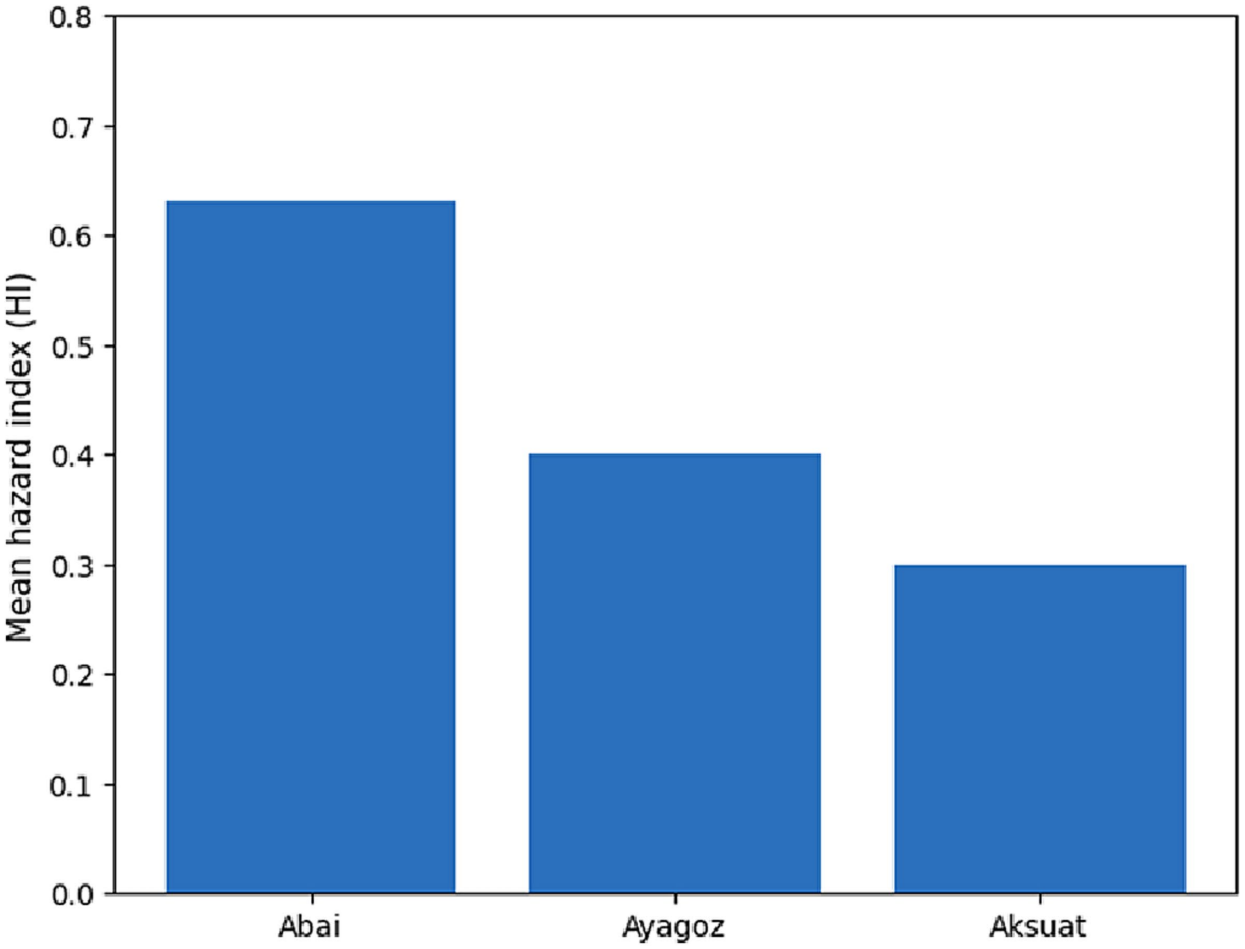
Descriptive district-wise mean hazard index values across meat categories. Bars represent arithmetic means of product-specific HI values and are shown as descriptive summaries only.

As shown in Figure 2, the arithmetic mean HI values across districts for different meat categories were 0.63, 0.40, and 0.30 for Abai, Ayagoz, and Aksuat, respectively. These values are presented as descriptive summaries of product-specific risk indices and should not be interpreted as independent exposure indicators. These findings clearly indicate a higher cumulative burden in the district located closest to the former nuclear test site.

Importantly, the fact that the concentration-based regulatory exceedance for Pb in milk did not translate into an HI > 1 under the adopted exposure assumptions must be interpreted with caution. These risk estimates apply strictly to the adult exposure scenario and should not be extrapolated to children, pregnant women, or high-consumption groups without separate exposure calculations that account for age-specific and physiological vulnerabilities. Consequently, the recurring Pb exceedance relative to the concentration-based limit warrants continued, rigorous veterinary-sanitary surveillance and target interventions, particularly within the Abai district.

Risk calculations were performed assuming an average daily meat consumption rate of 0.192 kg/day and 0.548 L/day for milk, which strictly correspond to the annual intake volumes of 70 kg/year and 200 L/year. All calculated THQ values for individual toxic metals were below the safety threshold of 1.0. Product-specific HI values ranged from 0.257 to 0.763, with the highest values observed for mutton (0.763) and beef (0.743) from the Abai district, and the lowest value for beef from the Aksuat district (0.257).

## 4 Discussion

Thus, the results indicate pronounced interdistrict heterogeneity in contamination levels, with a tendency toward higher concentrations of radionuclides and toxic metals in the Abai district and lower values in more remote districts. However, this pattern was not universal for all product types and contaminants, highlighting the influence of local factors, including pasture characteristics, feed sources, soil conditions, and contaminant migration pathways. Therefore, the observed pattern should be interpreted as a general spatial trend rather than as a strictly sequential gradient of decreasing contamination with increasing distance from the potential source.

Similar spatial heterogeneity has previously been reported in other agricultural regions affected by long-term radiological or anthropogenic contamination. Under such conditions, residual fallout, land-use patterns, and local environmental characteristics may continue to influence contaminant transfer along the soil—feed—animal—food product pathway even decades after the cessation or reduction of the primary source of contamination (17, 18).

### 4.1 Radiological characteristics and spatial heterogeneity of contamination

Overall, the findings indicate interdistrict spatial heterogeneity in the contamination of food products, reflected by relatively higher levels of radionuclides and toxic metals in the Abai district and lower values in more distant districts, such as Ayagoz and Aksuat. This pattern may reflect the influence of geographical proximity to the former Semipalatinsk Nuclear Test Site, historical contaminant deposition, and local natural, climatic, soil-geochemical, and agricultural conditions.

However, the observed tendency was not equally pronounced for all product–contaminant combinations. In some cases, values recorded in more distant districts were comparable to those observed in the Abai district or deviated from the expected decreasing sequence. Therefore, the results should not be interpreted as an ideally monotonic spatial gradient, but rather as a general trend of interdistrict variability in contamination levels. This is particularly important for territories with a long history of anthropogenic impact, where the current distribution of contaminants is determined not only by distance from the source but also by a combination of local environmental factors.

The radiological data showed that ^137^Cs activity in all analyzed meat samples remained far below the maximum permissible level of 200 Bq/kg. This indicates that, despite the possible detection of trace amounts of historically derived radioactive fallout in agroecosystems, current ^137^Cs levels in products of animal origin remain low under typical production conditions (16, 18).

The comparatively lower levels of ^137^Cs observed in poultry meat compared with beef, horse meat, and mutton may be associated with species-specific biological characteristics, differences in diet and housing conditions, and a shorter period of radionuclide accumulation in poultry. This finding is consistent with radionuclide transfer studies showing that the accumulation and retention of radioactive elements in farm animals depend on animal species, tissue type, and duration of exposure (7, 26).

The calculated annual effective dose values were also substantially below the recommended public exposure limit of 1 mSv/year. The corresponding estimates of excess lifetime cancer risk remained within a low-risk range, indicating limited radiological hazard associated with the consumption of the investigated meat products under current conditions. Comparable levels of radiological risk have been reported in studies of food chains in other environmentally vulnerable regions of Europe and Asia (24).

The contribution of ^137^Cs to the total dose from meat consumption was dominant. This can be explained by its relatively efficient transfer to animal muscle tissue, which represents the main edible fraction of meat products. In contrast, ^90^Sr has a stronger affinity for bone tissue, which limits its contribution to the dose associated with the consumption of muscle fractions. Thus, the radiological risk from meat products in the studied region was mainly determined by ^137^Cs, whereas ^90^Sr played a secondary role.

The observed interdistrict differences were likely influenced not only by distance from the former Semipalatinsk Nuclear Test Site but also by local soil and pasture characteristics. Parameters such as soil pH, particle-size and mineral composition, organic matter content, moisture conditions, vegetation type, and pasture-use practices may substantially affect the mobility of radionuclides and toxic metals, their bioavailability, and their subsequent transfer along the soil–plant–animal–food product pathway (7, 8).

Therefore, the spatial heterogeneity observed in the present study likely reflects the combined influence of residual contamination, local geochemical conditions, land use, and agricultural practices. Similar long-term heterogeneity has also been reported in other agricultural regions affected by radioactive or mixed anthropogenic contamination, where residual deposits, soil characteristics, and local environmental conditions continue to influence contaminant transfer decades after the initial source of exposure (15, 17, 18, 23, 27).

### 4.2 Chemical safety and toxic and potentially toxic metals

A critical finding of this study is that Lead (Pb) concentrations in whole cow’s milk consistently exceeded the regulatory maximum permissible concentration (MPC) of 0.1 mg/L across all evaluated districts, peaking at 0.272 ± 0.068 mg/L in the Abai district. To interpret these findings accurately, a clear methodological distinction must be made between regulatory compliance monitoring and deterministic health risk modeling. Regulatory limits (such as MPCs) are absolute safety thresholds established by law to ensure broad public health protection under any exposure conditions. Any exceedance is an unambiguous indicator of food safety non-compliance that demands rapid mitigation and institutional intervention.

Conversely, the calculated Target Hazard Quotient THQ_Pb_ = 0.55 and cumulative Hazard Index HI = 0.85 for the Abai district remained below the conventional screening threshold of 1.0. This mathematical discrepancy occurs because the deterministic risk model evaluates long-term chronic exposure using standard physiological assumptions for a healthy adult population (70 kg body weight, moderate consumption rates). While an HI < 1 suggests that immediate non-carcinogenic health effects are unlikely for average adult consumers, it must not be used to downplay or dismiss the regulatory exceedance.

Crucially, the current adult-centric model cannot be extrapolated to sensitive or high-consuming sub-populations. Children and pregnant women are uniquely vulnerable to chronic lead exposure due to distinct physiological and behavioral factors. Children exhibit higher gastrointestinal absorption rates for heavy metals, lower body mass, and are undergoing critical phases of neurodevelopment. In a scenario where the exposure parameters are adjusted for children-who typically consume more milk per unit of body weight-the THQ_Pb_ and integrated HI would highly likely cross the critical threshold of 1.0, indicating a clear non-carcinogenic health risk. The lack of age-stratified, localized consumption data for the Abai region prevents a reliable probabilistic risk estimation for pediatric cohorts, which remains a significant public health caveat of this research.

This interpretation is consistent with current food safety risk assessment practice, according to which contaminant concentrations, actual consumption levels, body weight, exposure duration, and toxicological reference values should be considered together (10, 22). Therefore, exceedance of a regulatory limit and a calculated HI below 1 are not mutually exclusive findings; rather, they reflect different levels of assessment: the former characterizes product compliance with safety requirements, whereas the latter characterizes the estimated health risk under a defined exposure scenario.

### 4.3 Integrated assessment of chemical and radiological factors in the international context

Populations in historically contaminated areas are exposed to complex mixtures of radiological and chemical hazards rather than isolated factors. The Abai region exemplifies this, combining the long-term radiological legacy of the former Semipalatinsk Nuclear Test Site (SNTS) with local geochemical heterogeneity and toxic metal transfer into the human food chain. Our findings reinforce the One Health framework by demonstrating the tight mechanistic continuum between environmental degradation, livestock health, and human dietary safety. The SNTS legacy acts as a dynamic ecological driver: persistent soil radionuclide ^137^Cs, ^90^Sr and toxic metals Pb, Cd transfer to pasture vegetation and are ingested by free-ranging livestock. Due to chemical similarities to essential ions-where ^90^Sr mimics Calcium in bone matrices and Pb targets divalent metal transporters (28, 39) -these contaminants cross epithelial barriers, bioaccumulating in animal tissues and partitioning into milk (29, 40). Therefore, ensuring food safety requires a shift from isolated end-product testing to integrated veterinary-environmental monitoring of pasture management and soil-to-plant transfer factors. In an international context, the post-contamination landscape of the Abai region shares striking long-term radioecological similarities with the late-phase recovery zones of Chernobyl and Fukushima (16, 18, 23, 24). Decades after the primary fallout, all three regions exhibit a substantial decline in acute atmospheric radioactivity, transitioning into a phase dominated by chronic, highly heterogeneous radionuclide transfer within agroecosystems.

Long-term monitoring in post-Chernobyl agricultural systems (e.g., the Polessie and Bryansk regions) revealed that ^137^Cs activity in milk remains variable and ecologically persistent (17, 18, 30, 37). This is driven not by fallout density alone, but by localized soil properties-such as low exchangeable Potassium K^+^, high organic matter, and acidic pH-which enhance Cesium bioavailability. Similarly, longitudinal testing in Fukushima demonstrated that while systemic food contamination dropped rapidly due to strict countermeasures, specific transfer pathways (e.g., forest-pasture ecotones) demand continuous monitoring (16, 31, 38). Our data align with this global paradigm: current ^137^Cs levels are safely below international thresholds, yet significant inter-district heterogeneity persists, proving that late-phase risks are driven by micro-geochemical factors and farming practices rather than a simple distance-decay model from ground zero (32, 33). Regarding toxic metals, international comparisons show that Pb and Cd distribution in livestock products is heavily influenced by soil composition, feed quality, and husbandry practices (10, 11, 15). Under pasture-based management, cattle, sheep, and horses ingest contaminants through both vegetation and incidental topsoil consumption. In contrast, controlled poultry feeding systems isolate birds from contaminated pasture, explaining the absence of a detectable ^90^Sr contribution in poultry meat. Consequently, the observed exceedances of Pb in whole cow’s milk constitute an independent chemical food safety concern requiring targeted monitoring, irrespective of radiological risk profiles. Methodologically, this study evaluated chemical and radiological components separately (using AED/ELCR for radionuclides and THQ/HI for metals), which is justified by their differing toxicological principles. However, this does not capture potential cumulative or mixed exposure effects. Future studies must operationalize mathematically combined risk indices, particularly for vulnerable cohorts (34, 35). In conclusion, the Abai region represents a classic late-phase anthropogenic impact zone where current radiological risks remain low, but spatial heterogeneity in contaminant transfer persists. Managing these risks demands comprehensive food monitoring that tightly integrates local natural, geochemical, and agricultural vectors (10, 11, 15, 16, 18, 23, 36).

### 4.4 Practical implications of the study

The main contribution of the present study lies in the integrated assessment of radionuclides and toxic and potentially toxic metals in several categories of products of animal origin obtained from districts located at different distances from the former Semipalatinsk Nuclear Test Site. Unlike studies limited to individual contaminants or specific product categories, this approach provides a more comprehensive characterization of the potential dietary burden to the population through the food chain.

The findings have practical significance for veterinary and sanitary surveillance and food safety assessment. They show that monitoring in areas with a history of anthropogenic impact should be not only regular but also territorially differentiated. Particular attention should be paid to districts where contaminant concentrations are consistently higher, as well as to product categories that show the greatest sanitary and hygienic relevance. From a practical perspective, the most important finding is the need for enhanced control of dairy products, especially whole cow’s milk, in which Pb concentrations exceeded the applicable regulatory limit. This demonstrates that even when calculated non-carcinogenic risk values are low, individual products may fail to comply with regulatory requirements and may require additional control measures.

In addition, the results confirm the need for a product-specific approach to monitoring. Meat, milk, and products from different animal species differ in the mechanisms by which radionuclides and toxic and potentially toxic metals accumulate. Therefore, food safety assessment should not be based solely on average territorial values, as these may mask elevated contaminant levels in specific products.

The risk assessment framework, including AED, ELCR, THQ, and HI, may be used as an additional tool for prioritizing control programs. This approach makes it possible to distinguish cases of regulatory non-compliance from scenarios of increased estimated health risk, thereby avoiding both underestimation of the problem and overinterpretation of individual concentration values.

In a broader context, the practical significance of this study lies in demonstrating the need for integrated surveillance at the interface of environmental protection, animal health, and public health. This approach is consistent with the principles of One Health and may be useful for developing regional food monitoring programs in areas with long-term radiological and chemical legacies.

### 4.5 Limitations of the study

The present study has several limitations that should be considered when interpreting the findings. First, the risk assessment was performed only for the adult population. Separate exposure scenarios for children, pregnant women, individuals with high milk and meat consumption, and other sensitive population groups were not modeled. Therefore, the obtained THQ, HI, AED, and ELCR values should not be directly extrapolated to the entire population without additional calculations.

Second, the exposure assessment was based on assumed consumption scenarios and standard coefficients rather than individual dietary data from residents of the studied districts. In rural areas, the actual consumption of locally produced foods, particularly milk and meat from household farms, may differ from national average values. This introduces uncertainty into the estimation of actual radionuclide and toxic metal intake.

Third, seasonal variations in contaminant concentrations were not assessed. Concentrations of radionuclides and toxic and potentially toxic metals in products of animal origin may vary depending on season, feed composition, grazing regime, pasture availability, water quality, and other factors. Therefore, single-time-point or time-limited measurements may not fully reflect annual exposure patterns.

Fourth, direct human biomonitoring was not conducted. Risk estimates were based on contaminant concentrations in food products and exposure models. Direct measurement of radionuclides and toxic metals in human biological substrates, such as blood, urine, hair, or teeth, as well as assessment of internal exposure using whole-body counting, was not performed. This limits the ability to confirm the actual dosimetric and toxicological burden among residents of the Abai region.

Fifth, the risk assessment was performed using a deterministic approach based on fixed mean concentrations and standard exposure parameters. Although this approach is widely used in food risk assessment studies, it does not fully account for variability in exposure among different population groups. The absence of probabilistic modeling, such as Monte Carlo simulation, limits the ability to quantify uncertainty and identify high-exposure scenarios.

Sixth, chemical and radiological risks were assessed separately. Under real-life conditions, the population may be simultaneously exposed to mixtures of contaminants, including radionuclides and toxic and potentially toxic metals. Potential additive, synergistic, or antagonistic effects of combined exposure were not quantitatively evaluated in the present study. Therefore, the interaction between chemical and radiological stressors remains an important direction for future research.

Seventh, carcinogenic risk from Pb and Cd was not quantified. Although these elements may have carcinogenic relevance depending on exposure route, dose, and toxicological framework, the present study was limited to non-carcinogenic dietary risk indicators for toxic and potentially toxic metals. Future research should include carcinogenic risk assessment where appropriate oral cancer slope factors and local exposure parameters are available.

In addition, the study was limited to selected categories of products of animal origin. Other dietary components, including plant-based foods, water, wild food resources, and processed products, were not considered. This may also affect the completeness of the assessment of total dietary exposure.

Thus, the present findings should be interpreted as an important but not exhaustive assessment of the safety of products of animal origin in the studied districts. Future studies should expand the assessment to include different age groups, seasonal monitoring, local food consumption data, probabilistic risk assessment methods, and combined exposure to radionuclides and toxic and potentially toxic metals. Such limitations are typical of comparable food risk assessment studies; however, addressing them would improve the accuracy and practical relevance of future monitoring programs (11, 21, 24).

## 5 Conclusion

Pronounced spatial differences in the levels of radionuclides and toxic and potentially toxic metals in products of animal origin were identified across the studied districts of the Abai region. In general, higher contaminant levels were more frequently observed in the Abai district, which is located closer to the former Semipalatinsk Nuclear Test Site, whereas lower values were mostly recorded in the more distant districts of Ayagoz and Aksuat. However, this pattern was not completely consistent across all product types and contaminants, indicating the influence of local soil-geochemical conditions, pasture management practices, feed sources, and species-specific accumulation mechanisms.

The radiological indicators suggest a low current level of risk for the adult population under the adopted consumption scenario. The activity of ^137^Cs in all analyzed meat samples remained below the maximum permissible level, and the calculated annual effective dose and excess lifetime cancer risk values did not exceed the recommended threshold levels. ^137^Cs was the dominant contributor to the dose associated with meat consumption, whereas ^90^Sr played a secondary role, particularly in animal muscle tissues.

The chemical analysis showed that particular attention should be paid to lead content in milk. Pb concentrations exceeded the established regulatory limit in all studied districts, indicating non-compliance of this product with food safety requirements and the need for enhanced control. At the same time, the calculated THQ and HI values remained below 1.0 under the adult exposure scenario. Therefore, exceedance of the concentration-based regulatory limit and the absence of exceedance of the calculated hazard index are not mutually exclusive findings: the former reflects the regulatory and sanitary significance of the problem, whereas the latter characterizes the estimated risk under defined assumptions regarding consumption rate and body weight.

Overall, the findings indicate that the current radiological and non-carcinogenic risks associated with the consumption of the investigated products of animal origin remain low for the adult population under the assumptions used. However, these findings should not be extrapolated to children, pregnant women, high-consumption groups, or carcinogenic endpoints without separate exposure assessment. Although calculated THQ and HI values remained below 1 under the adopted adult exposure scenario, the repeated exceedance of the regulatory Pb limit in milk requires continued veterinary-sanitary monitoring and should not be interpreted as complete absence of concern for sensitive population groups. Persistent spatial heterogeneity of contamination, repeated Pb exceedances in milk, and possible differences in consumption patterns among population groups confirm the need for continued monitoring. Particular attention should be given to dairy products, districts with higher contaminant concentrations, and potentially vulnerable population groups, including children and individuals with high consumption of locally produced foods.

Thus, the study supports the need for comprehensive, territorially differentiated, and product-specific veterinary and sanitary monitoring in historically affected regions. Such an approach, integrating radiological and chemical food safety assessment, is consistent with the principles of the One Health concept and may provide a basis for preventive decision-making at the interface of environmental protection, animal health, and public health.

## Data Availability

The raw data supporting the conclusions of this article will be made available by the authors, without undue reservation.
